# Tubal Pathologies and Fertility Outcomes: A Review

**DOI:** 10.7759/cureus.38881

**Published:** 2023-05-11

**Authors:** Amin-Florin El-Kharoubi

**Affiliations:** 1 Obstetrics and Gynecology, University of Oradea, Oradea, ROU

**Keywords:** pid, tubal patency, female fertility, fertility sparing treatment, tubal infertility

## Abstract

Anomalies of the fallopian tubes represent one of the most significant elements that might contribute to reproductive issues. They can be inherited or acquired; they are among the most important problems of the profession. Although there is much discussion regarding which therapies for each tubal disease are the most effective and result in the best long-term reproductive outcomes. During the evaluation of an infertile couple, certain anomalies of the fallopian tubes are frequently discovered. These abnormalities were thought, for a long time, to not have an influence on fertility; however, in recent years, researchers have discovered that they seem to play a crucial role in fertility problems. Couples in industrialized countries are postponing childbearing, which raises the risk of women developing tubal diseases before they are ready to become pregnant. These disorders may have a negative impact on a woman's ability to get pregnant. The goals of this study are to conduct research to gain a deeper understanding of the recent advancements that have been made in the field of tubal diseases and to carry out an evaluation of the medical conducts that have the best fertility outcomes.

We searched both Medline and PubMed, paying special attention to the most relevant articles that have been added to either database over the course of the last six years.

## Introduction and background

The World Health Organization identifies infertility as a public health concern. Infertility is characterized by the American College of Obstetricians and Gynaecologists as the inability to become pregnant after 12 months or more of unprotected intercourse for women under 35 and after six months for women over 35, Around 35% of infertility causes are related to females, with tubal factors contributing to approximately 20% of these instances [1]. Fallopian tubes are vital components of the female reproductive system due to their crucial role.

In vitro fertilization (IVF), developed over three decades ago, provides a solution for individuals facing infertility due to tubal problems. Nonetheless, treatment expenses remain elevated, and more sophisticated technologies and costly treatments have emerged over time [2].

The ideal strategy for patients with fallopian tube abnormalities who wish to maintain their fertility continues to be debated. Surgical procedures to evaluate tubal patency can occasionally uncover abnormalities with an uncertain connection to infertility. The latest advancements in diagnostic methods, clinical and surgical treatments for various fallopian tube abnormalities, and their effects on fertility are constantly being updated. This study's goal is to review the literature to gain insight into the most recent developments in the field of tubal abnormalities and to examine the medical approaches that provide the best fertility outcomes. A thorough search of Medline and PubMed was conducted for articles published up to January 5, 2023, with an emphasis on the most pertinent studies from the previous six years. Approximately 100 articles on tubal abnormalities were reviewed to gather high-quality information on the topic.

## Review

Tubal congenital anomalies and fallopian tube congenital malformation may be classified into three categories: 1. Total, Partial, or segmented absence, 2. Duplication, which can affect ostia and/or the tubes, and 3. Multiple lumina and diverticula. Since these minor tubal anomalies were not previously reported to have any effect on fertility, there aren't many studies on their prevalence in general. Subtle variations in tubal anatomy were found more commonly in infertile women [3-5]. These abnormalities are caused by the splitting of the cranial extremities of Müllerian ducts. Infertility results from the accessory tube being obliterated at the point where it connects to the main tube and the ova being wasted after being captured by the accessory tube [6]. The accessory tube can cause pyosalpinx, cystic swelling, torsion, and ectopic pregnancy; moreover, it is associated with endometriosis [7,8]. Due to the absence of any clinical signs, making a diagnosis of fallopian tube accessory ostium ahead of surgery may be very challenging, Some authors recommended surgical excision when these abnormalities were accidentally found in order to improve reproductive chances and prevent complications [6].

At laparoscopy, tubal diverticula are tiny, thin-walled out-pouches seen in the fallopian tube's ampullary section and tubal isthmus. Only a few reports of tubal diverticula have been published in the literature; it is more prevalent in women with endometriosis than in infertile patients, The distal end of the tubes may accumulate radiopaque contrast media during a hysterosalpingogram (HSG), which might indicate the existence of diverticula. the correlation of this pathology with infertility is still debated the limited studies have not yet succeeded in demonstrating the cause-effect of this pathology on infertility [9], and the result of IVF success for patients with no other found cause of infertility was significantly high [10,11].

Paraovarian and paratubal cysts

About 10% of adnexal masses consist of paraovarian and paratubal cysts, which are located between the ovary and the fallopian tube in the broad ligament. These cysts are relatively common, usually asymptomatic, and often discovered incidentally. They are thought to originate from mesothelium or remnants of paramesonephric (Müllerian) and mesonephric (Wolffian) ducts. Remnants of the paramesonephric duct, known as hydatids of Morgagni, often develop within the broad ligament instead of at the fimbriated ends of the fallopian tube. They are small serous fluid-filled cysts that are more frequently identified with the advent of endovaginal ultrasonography. Due to their small size of less than 2 cm, they are difficult to differentiate from ovarian cysts. Paraovarian cysts are simple, fluid-filled cysts, 1-8 cm in diameter, and generally asymptomatic. However, they may become clinically significant in rare instances due to their size and/or torsion. Distinguishing between ovarian and paraovarian cysts can be challenging [12]. The "split" sign, seen on ultrasound images, helps establish a diagnosis. It is characterized by the separation of a paratubal cyst from the adjacent ovary when pressure is applied using a transvaginal transducer.

Torsion and large cyst size require more urgent treatment, It is important to note that hydatid of Morgagni has been found in over half of patients with unexplained infertility, potentially acting as an obstacle for fimbria in picking up the ovum. Removal of these cysts can improve ovum pick-up and enhance fertility in patients without other causes of infertility [13]; their surgical resection leads to favorable results on fertility [14].

Pelvic inflammatory disease (PID)

PID, in the majority of cases, is linked to sexually transmitted infections like Chlamydia (C.) and gonorrhea. The uterus, fallopian tubes, and/or ovaries can all be impacted, and the infection often ascends from the lower genital tract. Estimates suggest that among women aged 35 to 44, 33.6% have experienced at least one episode of salpingitis, and 16.1% have experienced at least one episode of PID, while 10.7% have only experienced one episode of salpingitis without any additional PID episodes [15]. PID can result in scarring, adhesions, and partial or total occlusion of the fallopian tubes. This may lead to the loss of ciliated epithelial cells in the inner layer of the fallopian tube, impeding ovum transit and increasing the risk of infertility and ectopic pregnancy. Adhesions may also cause persistent pelvic pain [16]. PID can be acute, chronic, or subclinical and is often underdiagnosed. Symptoms of PID can be mild or insidious, and some women may not show any signs or symptoms, only realizing the issue when faced with infertility or chronic pelvic pain.

Salpingitis may not be visible on ultrasound, and when it is, it may appear as a swollen, convoluted fallopian tube or hyperaemic wall on color Doppler. Significant tubal distention is often not observed at this stage. Free fluid may be found in the Douglas pouch. Swollen tubes with enhanced mucosal appearance can be seen on CT and MR images with intravenous contrast. The 2011 UK national PID guideline states that recent-onset lower abdominal pain and localized tenderness on bimanual examination are sufficient for a diagnosis and initiation of treatment [17,18]. A prospective cohort study found that 10.8% of women with laparoscopically confirmed acute (non-tuberculous) salpingitis who attempted pregnancy were infertile due to post-PID tubal blockage [19]. PID is considered the main cause of tubal adhesions and acquired abnormalities, with early treatment being the key factor influencing the disease's outcomes and progression.

Tubo-ovarian abscess (TOA)

TOA represents a more advanced stage of infection and inflammation. These abscesses typically develop as a late consequence of PID and form a complicated infectious mass in the adnexa. Infectious pathogens initially ascend from a vaginal or cervical infection to the endometrium, then through the fallopian tubes and into the peritoneal cavity, where they form a walled-off mass. Peritonitis is often seen in related cases. TOAs can also develop due to the spread of infection from a nearby organ, most commonly the appendix, or in conjunction with pelvic organ malignancy [20]. In the past, over 20% of hospitalized PID patients were found to have a TOA, but with the introduction of new guidelines for the evaluation and treatment of sexually transmitted diseases by the Centers for Disease Control and Prevention in 2002, the incidence of TOA has decreased.

Historically, treatment for TOA involved a complete abdominal hysterectomy and bilateral salpingo-oophorectomy. Nowadays, a ruptured TOA is a surgical emergency while the management of an unruptured TOA is more controversial. Most studies report a success rate of 70% or higher with conservative management of TOA [21-23]. As most of these patients are women of reproductive age, hormonal function and fertility are important concerns. Intravenous antibiotics, combined with interventional radiology, such as transvaginal ultrasound-guided drainage, have shown good results for TOAs smaller than 5 cm. For TOAs larger than 5 cm, a laparoscopic approach is recommended [24], which can also preserve fertility in approximately half of the patients [25]. With medical treatment alone, there is a 3%-4% risk of rupture and potentially fatal peritonitis, whereas immediate laparoscopy has a success rate of 90%-100% [26-28]. Tubo-ovarian abscesses have a negative impact on ovarian function, with poor pregnancy rates observed following TOA [29]. Infertile women may be candidates for early laparoscopic intervention to maximize their chances of pregnancy after a future frozen-thawed embryo transfer [30,31].

Salpingitis isthmica nodosa (SIN)

The isthmic portion of the fallopian tube can develop a nodular swelling of up to a few centimeters in diameter, although it can also affect other parts of the fallopian tube. While the exact cause remains unknown, it is likely due to an acquired process. There are currently three proposed etiologies: infection, cellular invasion, and congenital malformations [32]. Studies have demonstrated that the outer membrane protein of C. trachomatis and/or high serum antibody titers are often present in the affected fallopian tube of women who previously showed histological evidence of salpingitis, suggesting a link between SIN and past Chlamydia infection. According to the non-inflammatory theory, SIN results from the expansion of the fallopian tube's inner layer, which eventually invades the mucosal wall. The congenital theory suggests that the tube-like glands are Wolffian rests. Most evidence seems to support an acquired cause [33,34]. Its prevalence in healthy, fertile women ranges from 0.6% to 11%, but it is significantly more likely to occur in cases of ectopic pregnancy and infertility [35]. Hysterosalpingography is the first line of investigation for infertility and is also an effective diagnostic method for salpingitis isthmica nodosa [36]. There is no conservative treatment for SIN. Fertility management for patients with SIN often involves assisted reproductive technology [35], with studies showing an 80% chance of live birth by the fifth cycle of IVF [37]. Salpingectomy is recommended for symptomatic patients when fertility is not a concern [38]. A few studies have investigated the surgical removal of the affected section as a treatment option. Following tubocornual anastomosis, the term pregnancy rate was 45% when the resected isthmus portion was less than 1 cm and 22% when it was more than 1 cm [39,40].

Hydrosalpinx

Hydrosalpinx is a relatively common condition that can be diagnosed alone or as part of a more complex disease process, such as pelvic endometriosis or PID. It occurs when secretions accumulate in the blocked fallopian tube lumen, preventing the secretions from escaping through the fimbriated end and entering the peritoneal cavity. PID is the most common cause of distal tubal occlusion and hydrosalpinx, with other less frequent causes including endometriosis, paratubal adhesions from previous surgeries, tubal tumors, and tubal ectopic pregnancies. Patients may be asymptomatic or experience frequent lower back or pelvic pain. On ultrasound, the enlarged longitudinal folds of the fallopian tube give hydrosalpinx its characteristic "cogwheel" appearance in cross-section. Differentiating hydrosalpinx from a multiloculated cystic ovarian tumor can be challenging when the tube has a large diameter (>10 cm); MRI can help clarify the situation due to its higher contrast and spatial resolution [41,42]

The treatment of hydrosalpinx poses a clinical challenge. Surgical options for individuals with fimbrial blockage include salpingostomy and fimbrioplasty, which are only suitable for small, thin-walled hydrosalpinx with healthy mucosa [43]. Evidence shows that the presence of hydrosalpinx reduces the overall success rate of assisted reproductive technologies in achieving pregnancies [44]. The presence of hydrosalpinx is associated with a lower total fertility rate and increased risk of ectopic pregnancy and miscarriage, possibly due to embryotoxic components in the hydrosalpinx fluid [45]. Treating hydrosalpinx increases the likelihood of pregnancy, regardless of the treatment modality used. Salpingectomy has been associated with higher rates of IVF live births, clinical pregnancies, and implantations compared to other hydrosalpinx treatments [46,47]. With conservative treatment, there is a reasonable chance of spontaneous conception [48,49]. The natural pregnancy rate within two years following neosalpingostomy was 50% for mild, 17.39% for moderate, and 15.6% for severe hydrosalpinx [50]. In fact, assisted reproductive technology and combined hydrosalpinx treatment result in a 61% cumulative pregnancy rate [48].

Tubal endometriosis

Tubal endometriosis is characterized by the presence of ectopic endometrial implants on the fallopian tubes. Microscopic tubal endometriosis is more common in individuals with endometriosis than macroscopic disease. If these implants bleed into the lumen, they can cause hematosalpinx. While the pathophysiology is not well understood, it is believed to be complex and dependent on the anatomical distribution of endometriotic lesions [51]. There are three histological classifications for tubal endometriosis: the most common type involves endometrial implants invading the tubal serosa or subserosa, affecting the peritoneal surface of the tubes. The second type, called "endometrial colonization," involves the invasion of tubal mucosa by endometriotic implants and is thought to have a unique etiology. The third type, "post-salpingectomy endometriosis," occurs after tubal ligation or salpingectomy in the remaining proximal section of the fallopian tubes [52,53].

Patients with tubal endometriosis may experience pelvic pain, but there is no clear association between the severity of symptoms and the stage of the disease according to the American Society for Reproductive Medicine classification [54]. Tubal endometriosis is associated with advanced stages of the disease [55] and is strongly linked to infertility [56]. Besides causing scarring on the tubes, which makes natural pregnancy unlikely, endometriosis also leads to inflammation in the pelvis. Increased reactive oxygen species (ROS) levels in endometriosis-induced inflammation and decreased antioxidant levels contribute to oxidative stress. This may result in inflammation-induced subfertility, as elevated ROS levels in the tubal fluid of endometriosis patients can negatively impact sperm, oocyte, and embryo viability [57].

A recent Cochrane review (Bafort et al., 2020) investigated whether surgery could improve the chances of achieving a natural pregnancy [54]. The analysis, based on moderately strong evidence from three randomized controlled trials primarily focused on intraperitoneal endometriosis, concluded that laparoscopic surgery increases the rate of viable intrauterine pregnancies. Another meta-analysis found that the pregnancy rate was significantly higher after laparoscopic surgery compared to placebo (OR 1.63; 95% CI 1.13 to 2.35) (Hodgson et al., 2020) [58]. Jin et al. (2014) reported a significant increase in live births among patients who underwent laparoscopic surgery (relative risk (RR) 1.52; 95% CI 1.26 to 1.84, four trials, 741 patients) [59].

For women in the early stages of the disease, artificial insemination with ovulation induction may be more effective in increasing the natural conception rate compared to expectant management [60]. A single randomized controlled clinical trial (RCT) of very low size showed a higher clinical pregnancy rate associated with extended GnRH agonist suppression prior to IUI (Kim et al., 1995) [61].

Ectopic pregnancy (EP)

EP occurs in about 1%-2% of pregnancies, Over 98% of ectopic pregnancies implant in the fallopian tubes. The exact cause of ectopic pregnancy is unknown, but most risk factors are associated with the likelihood of previous fallopian tube injury, including prior abdominal or pelvic surgery and PID. Currently, transvaginal ultrasonography and serum hCG level determination are used to diagnose unruptured ectopic pregnancy. Vaginal ultrasound is unable to determine the location of a pregnancy in a significant proportion of women. In terms of ectopic pregnancy location within the fallopian tube, 13% occur in the isthmic segment, 75% in the ampullary segment, and about 12% in the fimbrial segment [62].

After an ectopic pregnancy, the chance of having an intrauterine pregnancy drops to 52%, and the risk of a second ectopic pregnancy is 12.6% [63]. Ectopic pregnancy is one of the few medical conditions that can be managed expectantly, with medication or surgery. Surgical management is essential in the case of a ruptured ectopic pregnancy. Salpingectomy is the preferred procedure when the contralateral tube is healthy. Salpingostomy, where the ectopic pregnancy is dissected out of the tube and the fallopian tube is left in place, may be performed to preserve fertility on that side. Numerous systematic reviews have examined the outcomes of these two procedures on fertility in individuals with a healthy contralateral tube, but further research is needed to improve patient selection, surgical techniques, and follow-up intervals [64]. A recent meta-analysis showed that salpingectomy is clearly superior to salpingostomy in low-risk patients, as it reduces the likelihood of future spontaneous uterine conception. Salpingostomies may be underutilized in women with risk factors for tubal disease [65].

Clinically stable patients with minimal symptoms and a low amount of free intraperitoneal fluid may be candidates for expectant management, as well as those with an unruptured tubal ectopic pregnancy. Patients managed expectantly rather than with surgery appear to have higher fertility rates compared to those who underwent surgical intervention. Medical treatment should be chosen when expectant management is not possible. The reproductive outcome after ectopic pregnancy treated with methotrexate was 57.5% after one year and 66.9% after two years [66,67]. Women who wish to resolve the issue quickly, especially in the case of recurrent EP, may opt for surgical treatment [68].

Table 1 provides an overview of the most important articles regarding each pathology and the treatment available.

**Table 1:** An overview of the most important articles regarding each pathology and the treatment available

Tubal congenital anomalies	Partial or segmental absence solution: in vitro fertilization with good results [11]	Duplication recommended surgical excision [6]	Multiple lumina and diverticula are rarely associated with primary infertility [10]
	Due to the absence of any clinical signs, making a diagnosis of fallopian tube anomalies ahead of surgery may be very challenging. Some authors recommended surgical excision of accessory ostium in order to improve reproductive outcomes and prevent complications. The result of In vitro fertilization success for patients with no other found cause of infertility was significantly high in all tubal congenital anomalies [10]		
Paraovarian and paratubal cysts	Hydatids of Morgagni		Paraovarian cysts
	Distinguishing between ovarian and paraovarian cysts can be challenging. They act as an obstacle near the fimbria in picking up the ovum; extirpation of these cysts improves ovum pick-up and enhances fertility [13,14]		
Pelvic inflammatory disease	Nearly 20% of patients with pelvic inflammatory disease would experience infertility issues [15]. Grades of pelvic inflammatory disease have an adverse effect on In vitro fertilization outcomes. Receiving salpingectomy or not should be based on different grades of pelvic inflammatory disease [18]		Tubo-ovarian abscess: Once diagnosed, in order to preserve the ovarian function and resolve the case quickly, laparoscopic intervention is preferred over conservative management with a resolution of 90-100% compared to 20-87% [28,30]
	Pelvic inflammatory disease is considered to be the primary cause of tubal adhesions and acquired abnormalities [29]		
Salpingitis isthmica nodosa	There is no conservative treatment for salpingitis isthmica nodosa [37]. Tubocornual anastomosis can be attempted with a good result in fertility if the resected segment is less than 1 cm [39,40]. In vitro fertilization has good results, by the 5th cycle 80% of live birth		
Hydrosalpinx	Hydrosalpinx fluid has embryotoxic properties, which is why salpingectomy is recommended before In vitro fertilization [45]. Within two years following neosalpingostomy for hydrosalpinx, the natural pregnancy rate for mild, moderate, and severe hydrosalpinx was 50%, 17%, and 15%, respectively [48]. In fact, assisted reproductive technology and integrated hydrosalpinx treatment result in a 61% cumulative pregnancy rate [50]		
Tubal endometriosis	Additional to pelvic adhesions, endometriosis comes with an additional inflammation package in the pelvis [54]. In some cases, surgery can have a positive impact on pregnancy rates [56]		
Ectopic pregnancy	After an ectopic pregnancy, the uterine pregnancy rate was 52%. The repeat ectopic pregnancy rate was 12.6% [63]. Expectant conduct seems to have the most favorable results in the long term on fertility [68]. Salpingectomy has advantages over salpingostomy in patients classified as low-risk. Salpingostomy is preferable in women with risk factors for tubal disease [65]		

## Conclusions

In conclusion, anomalies of the fallopian tubes are a significant factor that can contribute to reproductive issues in women. These abnormalities were previously thought to have little to no impact on fertility, but recent research has highlighted their crucial role in fertility problems. With more couples in industrialized countries delaying childbirth, there is an increased risk of women developing tubal diseases before they are ready to become pregnant. Therefore, it is crucial to conduct further research to gain a deeper understanding of recent advancements in the field of tubal diseases and evaluate the medical conducts that result in the best fertility outcomes. This study underscores the importance of early diagnosis and treatment of tubal diseases to help women achieve their reproductive goals.
